# Promising Practices in the Frontiers of Quality Outcome Measurement for Intellectual and Developmental Disability Services

**DOI:** 10.3389/fresc.2022.871178

**Published:** 2022-04-28

**Authors:** Matthew Bogenschutz, Parthenia Dinora, Sarah Lineberry, Seb Prohn, Michael Broda, Angela West

**Affiliations:** Virginia Commonwealth University, Richmond, VA, United States

**Keywords:** intellectual and developmental disabilities (IDD), Home and Community Based Services, outcome measurement and valuation, advanced statistics, administrative datasets, co-researchers with disabilities, translational dissemination

## Abstract

Medicaid Home and Community-Based Services (HCBS) for people with intellectual and developmental disabilities (IDD) are vital for supporting people with IDD to live well in their communities, but there are not set standards for monitoring quality outcomes related to HCBS. In this paper, we propose promising practices for improving the quality of HCBS outcome measurement, based both in the literature and our own experience conducting an extensive U.S. state-level study. Specifically, we discuss: (1) using merged administrative datasets, (2) developing high-quality psychometrics that attend to ecological issues in measurement, (3) using advanced statistical analyses, and (4) creating immersive, user-friendly translational dissemination products. We conclude by suggesting what we see as important new frontiers for researchers to consider in order to enhance the quality of HCBS outcome measurement for people with IDD in the future.

## Promising Practices in the Frontiers of Quality Outcome Measurement for Idd Services

For people with intellectual and developmental disabilities (IDD) living in the United States, Medicaid Home and Community Based Services (HCBS) often provide access to vital supports for community living. While advocates, researchers, and policymakers have lauded the benefits of HCBS as a part of supporting deinstitutionalization and community integration for people with IDD, little empirical evidence exists to directly link HCBS service usage and expenditures to tangible outcomes for people with IDD or the overall service systems that administer HCBS.

In an era when the focus of policymakers has turned to the need for evidence to tangibly support public expenditures, outcome measurement in HCBS, one of the main mechanisms by which U.S. states provide supports to people with IDD in their communities, is increasingly important. Though there are controversies and critiques about use of evidence-based policymaking [e.g., (1, 2)], the use of evidence to measure HCBS outcomes and effectively disseminate those outcomes to policymakers and advocates is essential for compelling states to adequately resource HCBS as a means of promoting community living for people with IDD.

In this conceptual paper, we (1) outline a rationale for better outcome measurement in Medicaid HCBS for people with IDD, (2) examine four pressing challenges to better outcome measurement and how we have sought to address them, and (3) propose new frontiers for consideration in order to move HCBS outcome measurement into the future. We specifically focus on application to our work's approach to merging administrative datasets, using advanced statistical analyses, centering stakeholder voices, and creating immersive dissemination products. It is important to note that this paper is about a U.S. based study and suggestions may not readily apply in service and policy contexts outside the U.S.

## Background

### Quality Measurement in HCBS

HCBS enables people to live and work as part of their communities instead of residing in costly and segregated nursing homes or institutions (3). In fiscal year (FY) 2017, ~860,500 people with IDD in the United States used Medicaid IDD HCBS with estimated expenditures totaling $38.71 billion (4).

States that operate HCBS programs are required by the Centers for Medicare and Medicaid Services (CMS), the federal entity that governs Medicaid and Medicaid waiver programs, to measure and improve performance, assure that individual support plans meet the needs of waiver participants, and have effective systems in place to monitor participant health and welfare (5). States monitor compliance with these rules by using self-selected performance measures (6). Although flexibility in choosing performance measures has allowed states to be responsive to their individual needs and priorities, it has also created challenges with conceptualizing and measuring HCBS quality on a national level and for comparing outcomes across states (7, 8).

In recent years, discussions about HCBS quality and the need for national quality measurement standards have intensified (9). From 2014 to 2016, the National Quality Forum (NQF), contracted by the U.S. Department of Health and Human Services (HHS), convened a national stakeholder committee to develop measurement recommendations for monitoring HCBS quality (10). In September 2020, CMS issued a request for comment on a recommended standard measure set for Medicaid-funded HCBS (11). These recommended measures, organized based on quality domains identified by NQF, included the areas of service delivery and effectiveness, person-centered planning and coordination, choice and control, community inclusion, caregiver support, workforce, human and legal rights, equity, holistic health and functioning, system performance and accountability, and consumer leadership in system development (12).

### Stakeholder Input in IDD HCBS Quality Measurement

Stakeholder input, including gathering information directly from HCBS program participants with IDD, is fundamental to the CMS quality management strategy (13). In fact, CMS described their approach as “customer obsessed” with considerable participant outcome information integrated into their quality and performance standards (14).

To this end, multiple survey instruments have been used to assess participant experiences and outcomes associated with IDD HCBS. An early example was the Participant Experience Survey for people with intellectual and developmental disabilities (PES-DD). The PES-DD, which was designed to be administered in an interview format, measured IDD program participants' experience with HCBS services and focused on the four priority areas of access to care, choice and control, respect and dignity, and community integration/inclusion (15). A valid and reliable cross-disability HCBS participant survey was later created, which obtained the Consumer Assessment of Healthcare Providers and Systems (CAHPS) trademark and the NQF's endorsement (16). This tool, the HCBS CAHPS, includes questions about access to needed services, providers, case managers, choice of services, medical transportation, personal safety, community inclusion, and empowerment (17).

At the same time, measures outside of CMS were developed to assess user perception of IDD HCBS quality. For example, the Council for Quality and Leadership (CQL) constructed the Personal Outcome Measures (18) and the Human Services Research Institute (HSRI) and National Association of State Directors of Developmental Disability Services (NASDDDS) co-developed the National Core Indicators (19). Measures from the NCI have since been endorsed by the National Quality Forum (20). Both of these surveys are widely used in the United States to measure personal outcomes for people with IDD, including choice, health, safety, community participation, relationships, rights, and employment. The POM is often used to assess service provider quality, while the NCI is primarily used to assess the quality of state-level IDD service systems (19, 21).

### Aim and Scope of Paper

Considering the large outlay of public funds and the centrality of HCBS in the lives of many people with IDD and their families, improving the measurement of HCBS outcomes is an essential step toward monitoring system quality across the United States' myriad approaches to disability service provision. In addition, expectations of centering the experiences of people with IDD in the outcome measurement process have become increasingly prominent. Against this background, the current paper outlines four important emerging frontiers in HCBS outcome measurement, each punctuated by real-life applications from our work. We conclude by proposing additional actions that may be taken to improve HCBS outcome measurement and quality assurance in the future.

## Methods

The present article uses the example of one research team's experience, working to improve the quality of HCBS outcome measurement in their state. The research team members come from five academic disciplines (public policy, social work, education, evaluation, and rehabilitation counseling), have a variety of expertise (disability policy, advanced statistics, data management, etc.), and bring a wealth of lived experience as self advocates, family members, allies, service providers, and researchers. The research team has been assembled since 2016, when they began working on a pilot to merge three major datasets (as described below) in order to help policymakers in their state's DD agency and Medicaid agency come to a better understanding of how Medicaid HCBS expenditures related to important life outcomes for people with IDD. Over time, the research team's experience and approach has evolved, leading to the observations presented in this article.

### Overview of Virginia Costs and Outcomes Project

Much of this paper is based on the author's experience conducting their Virginia Costs and Outcomes project, which will be described in this section. Broadly, the Costs and Outcomes project is meant to help state-level policymakers, advocates, and other researchers understand how HCBS service expenditures relate to personal and system-level outcomes for adults (age 18+) with IDD, while accounting for individual support needs. Since 2015, we have been working on this project in stages, as described below. Before describing the phases of our project, however, it is necessary to emphasize the importance of careful pre-planning before endeavoring to look at HCBS outcome measurement in progressive new ways.

First, prior to starting our pilot work, there had been extensive relationship building with state DD and Medicaid agencies, which ultimately facilitated access to important datasets. We have continued to maintain close relationships with these agencies as our work has progressed, disseminating white papers for our state stakeholders, briefing them on the project process, and engaging them for input about specific research questions to pursue. We also had to think proactively about matters of research ethics, especially around using administrative data for research purposes. This included inserting explicit statements on the state DD agency's consent documents before we could use administrative data for our purposes, and working with our university's institutional review board to navigate the ethical oversight and review process for a complex administrative dataset that contained potentially sensitive data. Finally, our pre-work required strategy, particularly around our data management and integration process. We needed to ensure that we could link our key datasets at the level of the individual service user with IDD, which we accomplished by including a unique identifier that could be present on all of our data sources. The three datasets we use in our work are as follows:

#### Medicaid HCBS Expenditures

Furnished to us the state's Medicaid agency, Medicaid HCBS expenditures capture the array of long term services and supports (such as residential, employment/day program, and many other smaller services that people with IDD use long-term). Every HCBS user in Virginia has a Medicaid record.

#### SIS-A

The state's DD agency allows us to use SIS-A data, which the state uses with every HCBS user in order to assess their support needs. Every HCBS user in Virginia has a SIS-A on record.

#### NCI-IPS

The state's DD agency, which provides this dataset, randomly selects about 800 HCBS users annually to participate in the NCI-IPS, which measures a wide array of factors related to service usage, personal outcomes, and system-related outcomes. The annual sample of NCI-IPS users provides the base sample for our work and we obtain and merge SIS-A and Medicaid data based on the presence of a person's NCI-IPS record.

Second, our initial pilot work, funded by the Association of University Centers on Disability (AUCD) took place in 2015 and 2016. In this work, our main aim was to establish the feasibility of creating a large combined dataset from three sources with IDD-specific information merged at the individual level: (1) the state's Medicaid HCBS expenditure data for adults with IDD, (2) the state's data from the National Core Indicators - In Person Survey (19), and (3) the state's data from the Supports Intensity Scale-Adult version (22). The Medicaid HCBS data provided us with information about state expenditures per person on a plethora of services and supports, including various types of residential services, day habilitation and employment support services, respite, and case management. These data were available at a minute level of detail, so it was helpful to bundle them into usable units for analyses. The NCI-IPS provided a variety of outcome variables related to health, health service access, social inclusion, relationships, work or other day activities, choices, and rights that we have used as outcomes in our work. The SIS was useful to explore support needs, both overall, and in more specific domains such as medical support needs or behavioral support needs. Results of our pilot are available in Dinora et al. (23), and include findings about patterns of association between extraordinary medical and behavioral support needs and costs and some surprising findings related to residence type and community inclusion.

Third, following our successful pilot, we secured a 3 year research grant from the National Institute on Disability, Independent Living, and Rehabilitation Research (NIDILRR) to merge the same three datasets for an additional 2 years, and to link those two cohorts of data to begin the construction of a longitudinal linked dataset. As part of this work, we focused on improving psychometrics, specifically by attending to ecological issues that have troubled prior HCBS outcome measurement endeavors, and gave additional focus to the use of advanced statistical analyses.

Finally, in the current fourth phase of our project, funded by a NIDILRR grant running through 2024, we are adding two additional years of data to our merged dataset with linked cohorts, which will enable us to begin exploring the effects of the COVID-19 pandemic on HCBS outcomes and expenditures. In this phase, we will also be merging a fourth dataset, Medicaid managed care medical encounter data, which will help us understand how the frequency and intensity of medical service usage plays a role in personal and system outcomes for people with IDD. In this phase, we are also placing additional attention on stakeholder involvement and translational dissemination, and will have access to the NCI-IPS COVID-19 supplement, which provides information about the impact of the pandemic on people's lives.

### Identification of Promising Practices

In preparing to write this manuscript, the project team met to reflect on our experiences with the Virginia Costs and Outcomes project over the past seven years. Our aim was to identify the principal contributions of our work relevant to HCBS, with particular attention on what others may be able to learn from our work, how our progress was supported by prior research, and also where we still feel our project can grow in the measurement of HCBS outcomes within our context.

The vignettes in the promising practices section to follow are the result of that reflective discussion session and focus on four main aspects of our work that we believe are relevant to a wider audience: (1) using merged administrative datasets to improve HBCS outcome measurement for people with IDD, (2) developing high-quality psychometrics that attend to important issues of data and service system ecology, (3) using advanced statistical analyses, and (4) developing engaging, user-friendly dissemination products. For each of these topics, in the sections below we give a brief overview of the literature on the topic, before sharing a synopsis of our experience, which we hope will serve as a starting point for others to consider when engaging in their own efforts to improve HCBS outcome measurement.

## Promising Practices

### Using Merged Data Sources

The best HCBS outcome measurement requires high quality data that are capable of answering questions relevant to policymakers and advocates. However, the quality of data that has traditionally been used to track HCBS outcomes for people with IDD has been challenged by a number of factors, including inability to match fiscal and personal outcome data, and difficulty constructing robust datasets specific to people with IDD.

A number of authors [e.g., (23–25)] have called for better use of large administrative and linked datasets in the IDD field in order to generate a more nuanced portrait of the factors that may promote or inhibit particular outcomes. Though merging administrative datasets has been rare to date, there have been efforts, for example, to merge smart home and wearable technology data with Medicaid data to help understand safety and other personal outcomes for people with IDD (25). Despite such efforts, a national workgroup of leaders in the IDD field recently conducted an assessment of the potential to use administrative data to better understand health outcomes for people with IDD, including by potentially merging datasets, and concluded that current opportunities are limited, in part due to challenges in harmonizing definitions between datasets (26).

The use of administrative datasets to enhance the quality of outcome measurement in HCBS for people with IDD has other limitations as well. After examining several population-based datasets, Havercamp et al. (24) concluded that most datasets had severe limitations, related both to difficulty specifically identifying people with IDD in the dataset (IDD is conflated with other conditions as “cognitive impairment,” for instance), and to inadequate inclusion of people with IDD in population-based survey sampling. Wagner et al. (25) noted that many IDD-specific datasets are not robust enough to conduct advanced analysis, providing another significant barrier to using extant datasets to enhance the quality of HCBS outcome measurement for people with IDD.

#### Application to Virginia Costs and Outcomes

To begin to address some of the known challenges with using administrative and secondary datasets in outcome measurement, the Virginia Costs & Outcomes project endeavored to merge three major IDD-specific administrative datasets at the individual level. UsingVirginia's data from the National Core Indicators-In-Person Survey, the Supports Intensity Scale-Adult Version, and state Medicaid claims we created a large dataset, merged at the individual level and integrated across multiple annual cohorts, to create a robust randomly sampled dataset of people with IDD. The randomness in the sample comes from the state's NCI-IPS sampling method, which contacts about 800 randomly selected HCBS service users annula to solicit participation. We then merge in Medicaid expenditure data and SIS-A data for users with a valid NCI-IPS on record, since all HCBS users have those two datasets available (thus, our sample is bounded primarily by the availability of NCI-IPS records). With this dataset, we have been able to simultaneously account for two of the major drivers of service planning for adults with IDD: the need to carefully steward public funds, and the need to continuously monitor and improve outcomes for people with IDD who use HCBS.

Despite the success in being the first known team to successfully merge these three major datasets, we have continued to find that not all of our most important questions can be answered. Therefore, we are working with state partners in Virginia to obtain and merge Medicaid Managed Care acute encounter data, which will allow for a more granular understanding of healthcare utilization patterns and how they relate to outcomes. Additionally, we are exploring potential opportunities to layer additional, smaller datasets into our analyses, such as records for critical incidents, which would enable us to understand how outcomes and HCBS expenditures are affected after a person with IDD experiences a major traumatic event (e.g., abuse, injury, hospitalization, etc.).

All of this suggests that there are a multitude of possibilities to pursue in terms of merging extant administrative datasets, which each have some utility individually, but which hold significantly more potential for helping us understand HCBS outcomes when merged. In our experience, however, significant work needs to be done before any mergers take place, so researchers may wish to consider merging datasets as a years long investment before fruitful results emerge. Relationships must be built with state agencies, people with disabilities, and their families, processes for ethical compliance must be established, merger processes and unique keys to guide construction of the dataset need to be created well in advance, and a team with specialized skill sets must be assembled. It is also worth noting that conditions need to be right for open collaboration with state agencies, and often a defined policy window will open to facilitate collaboration. In our case, this window opened largely due to a consent decree between the state and the U.D. Department of Justice that was transforming much of the DD system in Virginia, including HCBS. If researchers can make significant initial commitments of time, however, the potential for merged datasets to transform outcome measurement for HCBS for people with IDD is significant.

### Developing High Quality Psychometrics

HCBS programs are influenced by both federal and state policy. Therefore, it is essential that we develop measures that can be used in both federal and state contexts. We will summarize our efforts to develop measures for tracking wellness and social outcomes across both federal and state-level ecological contexts.

The need for high-quality, psychometrically sound measurement tools in the IDD field has been well established, as mentioned previously (7, 8). A 2013 review of quality of life assessments for people with intellectual disabilities found that most of the identified instruments were not well validated (27). While most scales reported good to excellent validity, the majority did not report validation with people with varying levels of ID, floor and ceiling effects, or the factor structure of the scale (27). Similarly, Townsend-White and colleagues (28) reported that most quality of life measures had not been replicated and had only been validated by the developers.

Shogren (29) called for researchers to go beyond controlling for contextual factors to actively considering the role of political, cultural, and individual factors in quality of life outcomes for people with IDD. Prior literature on wellness and social outcomes for people with IDD has established the importance of considering the ecological context in which people use services. For example, research using the NCI-IPS found that the state in which people lived was a significant predictor of everyday and support-related choice (30). Similarly, Lu et al. (31) analyzed Medicaid claims data and found significant differences between states in level of adherence to diabetes care guidelines.

Other researchers have specifically examined the impact of state-level policy on outcomes for people with IDD [e.g., (32, 33)]. Sannicandro and colleagues (33) used advanced analytic techniques with a large administrative dataset to explore predictors of employment for adults with IDD. The authors found that people who participated in postsecondary education and lived in states with a higher employment rate for people with disabilities had better employment outcomes than people with the same level of education living in states with lower employment rates (33). Additionally, people who lived in states where a higher percentage of people with IDD were served by vocational rehabilitation had better employment outcomes than people who lived in states where fewer individuals were served (33). These findings reinforce the idea that state political and economic factors impact outcomes for people with IDD.

#### Application to Virginia Costs and Outcomes

Early in the Cost and Outcomes project, we found that most previously established measurement scales that had been developed from NCI-IPS variables did not perform well with our state's data, potentially due to the ecological issues discussed above [see (34, 35)]. Based on this poor statistical fit of previously developed scales to our data, we decided to develop new scales on our own. Our goal was to create variable clusters from the NCI-IPS that were statistically sound both in our state and using the NCI-IPS national dataset. To date, our team has used NCI-IPS data to create and test two scales: one to measure personal opportunities outcomes and another to measure wellness factors.

Our work began by using Virginia's merged NCI-IPS cohorts from 2017 and 2018 (total *n* of 1,608). Items from the NCI-IPS were initially selected based on their face validity to the relevant construct (wellness or personal opportunities) then examined using polychoric correlations to determine the strength of association between variables. Finally, confirmatory factor analysis (CFA) was used to test various factor structures for the models. This step is noteworthy, given that most scales identified by Li and colleagues (27) did not report testing multiple factor structures for their final model. Our preferred model for wellness included three variable clusters (mental health, behavioral support needs, and cardiac health indicators) and our preferred personal opportunities model contained four clusters of variables (relationships, community participation, rights, and daily choices).

Because we wanted to avoid the ecological issues that have been observed with previous measures developed from the NCI-IPS, we did not want to simply proceed with analyses based solely on state-level data, which may or may not apply in a national ecological context. To address this need, we obtained the NCI-IPS national dataset for 2018 from HSRI and NASDDDS. With their permission, we tested the fit of the two models we developed in our state data on the national dataset to see if they remained statistically sound. Finding good model fit in the national dataset, we tested the models as outcome variables in a series of linear regressions to check their utility and predictive validity. A full accounting of our methods and results may be found in Bogenschutz et al. (36) and Prohn et al. (37).

By using a rigorous method to develop scales to measure key HCBS outcomes for people with IDD and then testing those scales in both state-level and national datasets, we attended to the ecological challenges that have often troubled HCBS outcome measurement and attended to concerns raised by Li and colleagues (27) about statistical rigor in IDD measure development. In doing so, we created measures that have utility both to monitor our state's progress in achieving outcomes, and the ability to look at important outcomes for the nation as a whole.

### Using Advanced Statistical Analyses

The way we think about data analytics is shifting rapidly. Researchers in the field have been calling for use of more advanced analytic methods for some time, in a variety of applications such as using algorithms to identify people with IDD in population based or administrative datasets (38), innovating by using state or local level administrative datasets in novel ways (26), or using artificial intelligence in disability research (39, 40).

This last innovation, use of artificial intelligence in IDD research may have the power to be particularly transformative. For instance, while typical statistical methods commonly used in the IDD field are deductive, and therefore subject to the biases of past theory and literature that guide researcher's development of questions to be tested, machine learning is inductive, and driven entirely by the data. Although the potential for bias still exists due to flaws in datasets (especially when using historical data to predict present-day outcomes), the application of machine learning (and related methods such as propensity score matching) in the IDD field could potentially transform our evidence base for policymaking and advocacy, by generating truly data-driven evidence to support HCBS outcome measurement and system transformation.

Though tremendous potential for the use of artificial intelligence and machine learning methods in HCBS outcome measurement exists, so, too, do controversies. In some fields of social science research, most notably criminal justice (41), machine learning has come under scrutiny for potentially enabling the persistence of racial bias in, for example setting bail or determining eligibility for parole, since historical, racially biased samples, have sometimes been used to predict current outcomes. For IDD researchers to use large datasets ethically to help us better measure HCBS outcomes, we will need to find or create large datasets that more adequately represent the experience of HCBS users with IDD, avoid the use of historically biased datasets, be fully transparent about the predictive algorithms being used, and intentionally include the voices of HCBS users with IDD in our study design, implementation, and dissemination processes.

#### Application to Virginia Costs and Outcomes

In our work, we have employed machine learning to explore patterns of employment and day service utilization outcomes for HCBS users with IDD. To do this, we obtained the entire national NCI-IPS dataset for 2018 and constructed eight empirically-derived profiles of employment and/or day program participation that commonly occurred in the NCI-IPS dataset. Then we used all other variables from the NCI-IPS to train and test an algorithm to predict those eight employment and day program status outcomes. In order to avoid potential bias from past datasets, we did this by training the algorithm based on a randomly selected 80% training sample from the full dataset, and then testing the algorithm against the remaining 20% holdout sample. We tested both classification tree and random forest models, finding best fit based on the random forest algorithm. A full accounting of our procedures may be found in Broda et al. (42).

Our algorithm successfully predicted employment and/or day program participation outcomes with excellent accuracy (92% on the training sample, 82% on the holdout sample). Based on our analysis, the strongest predictors of employment and day program participation were (1) having a goal for employment in one's individual service plan, (2) having volunteer experience, and (3) being able to make one's own daily choices. This study was among the first in the IDD field to examine HCBS outcomes with machine learning, and showed both the feasibility and the practicality of doing so, since the results suggested that employment outcomes may be amenable to improvement with common-sense policy shifts.

### Creating Immersive, Accessible Dissemination Products

Outcomes research has a fundamental application to the lives of people with IDD and their families. It can also be a valuable tool for decision-makers when making IDD system investments. Whether at the “person-referenced level” (i.e., quality of life, self-determination) or at the “system-focused level” (i.e., characteristics of the system, services provided), outcomes research can provide valuable information to help inform decision making and service planning (43–45).

However, an ongoing challenge is the availability of clear and accessible information in formats that work best for people with IDD, families, and system-level decision makers (46–48). How we use language, image, audio, and video to convey research findings is a critical consideration when trying to enhance understanding and utility for stakeholders (49).

Social media tools such as Facebook, YouTube, Instagram and TikTok have become an increasingly common way that researchers communicate findings to constituencies (50). In the US, seven in ten people in the general population use some form of social media (51). Just like the general population, people with IDD reportedly are regular consumers of social media (52, 53). With social media there are concerns to consider such as acces, safety, accessibility and availability of support, possible misunderstandings of cyber etiquette, and communication and literacy skills (54). However, social media can be a powerful tool available to researchers to reach important stakeholders.

Additionally, for IDD outcomes research, making findings accessible and actionable to national and state IDD system managers is critically important. Despite this, often there is a considerable gap between researchers and policymakers when research is not clearly and expeditiously translated (2). One strategy with particular promise is distilling primary findings into a brief or summative format. Briefs, that summarize complex information in an accessible format, have been shown to be an effective tool for facilitating the use of research findings in policy decisions (55, 56)].

#### Application to Virginia Costs and Outcomes

Even the best outcome measurement is worth little unless it reaches policymakers and advocates in an understandable and actionable form. To that end, we have dedicated effort to the use of social media (Facebook Live events, TikTok videos, etc.) to translate complex findings into accessible and immersive products. These social media events regularly reach thousands of people with IDD and their families. Likewise, we have created easy to follow briefs and white papers that decision makers can use to drive program development and implementation in our state.

Through social media we have reached and engaged with new audiences that have posed specific questions about how our research can be used for real-life decision-making. We also are exploring how tools like TikTok, which generally attract a younger audience, can be employed to create conversations with youth as they enter service systems and bring with them new values and expectations about what they want from HCBS and how HCBS can support them to live good lives. Though use of TikTok has become more common among older users, we have also disseminated via Facebook and Instagram, in order to appeal to a broader spectrum of social media users.

We routinely engage with key stakeholders in the quest to get the right type of information to the right people in an accessible and useful way. The self advocate on our research team works directly with a statewide alliance of people representing a number of IDD advocacy groups across the state. They meet regularly to talk about ways that our research can help meet their needs, and they respond to our ideas and findings in a continual feedback loop. Using evaluative strategies, we continue to learn about what people want and need and recognize that flexibility is paramount, as information needs routinely evolve and change and are best addressed when customized for specific audiences (57).

## Next Frontiers

We are proud of our work to date in the Virginia Costs and Outcomes project, and have seen the impact that the above practices can make in the improvement of HCBS outcome measurement and monitoring for people with IDD. Still, we are continually looking for ways to improve, and the items in this section represent ways in which our team, as well as the field of IDD researchers generally, can continue to innovate to improve the quality of HCBS outcome measurement.

### Centering of Lived Experience

“Nothing about us, without us,” a central adage in the disability community, asserts that concerns that are integral to the lives of people with disabilities must be grounded in the voice of lived experience. This is especially true for research. People with disabilities are primary stakeholders in disability research, either as participants or as recipients of the policies and practices that are shaped by research findings (58). Despite this, people with disabilities, particularly people with IDD, have often been excluded from meaningful participation in research (59–61).

Integrally involving people with IDD in every aspect of the research process has demonstrated benefits. It can result in more relevant research questions grounded in lived experience; data collection methods and protocols that have greater feasibility, more nuanced and informed analyses of data, and improved dissemination strategies that reach end-users (62, 63). The “how” is where it can get more challenging. Co-researchers with IDD have reported challenges with securing needed accommodations to fully contribute to research design and development and have experienced power differentials with other researchers that affect their full participation (64–66).

We, as a field, need to continue work in partnership with people with disabilities so that every stage of the research process is infused with the voice of lived experience. Additionally, our findings must be authentically and accessibly communicated to people with disabilities and their families. Research that is focused on outcomes for people who use community-based services should be a tool that has utility for state or national decision-makers and in planning meetings where decisions are made about which services and supports would work best for people with IDD.

Our primary stakeholders, people with IDD, can be incredible assets to outcomes research in supporting these efforts. We must continue to support and strengthen inclusive research teams so that our research can have the greatest utility, reach, and impact.

### Scaling and Testing in Other States

Although promising in many regards, our work is limited by its narrow geographic scope, being confined to just one state. Because state systems vary widely, and since state-level policy and program changes may occur in a particular state but not in others, it is very important to take what we have learned in the Virginia Costs and Outcomes studies and apply it to other states. Doing so would help policymakers, researchers, and advocates come to a better understanding of how HCBS outcomes vary as a function of the policy environment in each state, and would help to gauge the quality of HCBS outcomes within a large national context.

Merging Medicaid HCBS expenditure claims, the SIS, and the NCI-IPS has been a productive exercise in HCBS outcome measurement for our team and for key stakeholders in our state, who have contributed to and benefitted from the work. Plans are currently underway to engage a similar process to merge the same datasets in five additional states, which we believe is an important step toward scaling our data integration method and eventually testing it in additional states. It will also be an opportunity to test our measurement scales for wellness and personal opportunities in other states in order to continue to address ecological issues in HCBS outcome measurement for people with disabilities that have posed such challenges in the past.

Scaling and testing in other states will likely take time and planning, as we have learned from our work. For instance, building relationships with state DD and Medicaid agencies is an ongoing process, developing procedures to embed a matching variable on all datasets to be merged takes coordination with state agencies, managing informed consent issues requires advanced planning and collaboration with ethics review boards, and data sharing agreements can take considerable time to secure. Researchers and state DD service managers in other states would be well served to plan longitudinally before undertaking a data merger process, but if such planning can be done intentionally, the scaling and testing of our (or similar) procedures for merging administrative datasets stands to be transformative for HCBS outcome monitoring for people with IDD.

### Translating Findings to Policy Action

Our greatest hope for our work, especially the work to longitudinally merge major administrative IDD datasets, is that it will provide a tool for state policymakers to use to both monitor the IDD service system in our state and to make fiscally responsible improvements to the HCBS system that will support high quality outcomes for people with IDD. Eventually, it is our hope that other states will see such impacts as well. In short, it is our hope that our work will help provide a solid empirical foundation for evidence-based policymaking.

Evidence-based policymaking is, however, unlike evidence-based medicine. Whereas, evidence-based medicine is premised on taking prudent action based on science from carefully planned clinical trials, evidence-based policymaking relies as much on emotion as it does on the rationality of empirical evidence (1). Policymakers and researchers come from different cultures, where policymakers often lack the technical knowledge to read and digest research reports that they often must act upon quickly as a policy window opens, and researchers often do not have the time, resource, or skill to distill technical findings in a meaningful way on tight timelines, leading to a disconnect between research evidence and policy making (2).

Given this disconnect between research evidence and policy making, in our continuing work, we are endeavoring to make greater investments in creating timely, short, and accessible bits of information that are actionable by policymakers. It is our intention that these pieces of information will also be accessible to advocates who influence policymakers, as we have been, and will continue to disseminate them via a variety of social media platforms as well as to advocates and policymakers directly. By making our findings accessible, actionable, and briefly summarized, we are hoping to bridge the research/policy gap, while continuing to conduct research based on innovative analyses and robust merged datasets that illuminate HCBS outcomes for people with IDD. Increasing use of personal narratives that use lived experience of people with IDD and their families to illuminate our empirically derived findings is also on our team's dissemination agenda. Although the effectiveness of narrative-based policy advocacy is not entirely clear (67), it is very much in line with our commitments to center lived experience in our work, and we are hopeful that it will be impactful in bringing voice to empirical findings.

## Conclusion

Medicaid HCBS provides essential services and supports to help people with IDD live well in their communities, and high quality outcome measurement is crucial to the process of continuously improving HCBS. By looking to promising practices from the field, such as using merged administrative datasets, addressing ecological issues in measurement, and engaging advanced statistical analyses, researchers can contribute to the enhancement of HCBS outcome measurement. Bringing the lived experience of people with IDD and their families directly into the research process, both as co-researchers and as consumers of accessible research results on HCBS, is also essential, as bringing lived experience to the forefront may be highly effective in the evidence-based policymaking process to strengthen and expand high quality HCBS services and supports.

## Author Contributions

MBo: led conceptualization, writing of most sections, editing, coordination, and core member of the research project being discussed. PD: wrote three segments of the manuscript, contributed substantive feedback on others, contributed to conceptualization of article, and serves as PI of the project being discussed. SL: wrote one segment of the manuscript, gathered and summarized literature, substantively edited, contributed to article conceptualization, and core member of the project being discussed. SP: contributed to manuscript throughout, critically reviewed earlier version of the manuscript, contributed to article conceptualization, and core member of the project being described. MBr: substantively contributed to three of the “promising practices” sections, provided substantive feedback throughout, provided detailed line editing, contributed to article conceptualization, and core contributor to the project described. AW: provided substantive feedback and edits throughout, aided in the conceptualization of article, provided self-advocate insights into how the article is presented, and core member of the research team for the project described. All authors have reviewed and given approval for submission of the manuscript.

## Funding

This work was funded by the National Institute on Disability, Independent Living, and Rehabilitation Research *via* a grant to the Partnership for People with Disabilities at Virginia Commonwealth University [Grant Number 901FRE0015-02-0].

## Author Disclaimer

The views expressed herein do not necessarily represent those of the funder.

## Conflict of Interest

The authors declare that the research was conducted in the absence of any commercial or financial relationships that could be construed as a potential conflict of interest.

## Publisher's Note

All claims expressed in this article are solely those of the authors and do not necessarily represent those of their affiliated organizations, or those of the publisher, the editors and the reviewers. Any product that may be evaluated in this article, or claim that may be made by its manufacturer, is not guaranteed or endorsed by the publisher.

## References

[1] Cairney P Oliver K. Evidence-based policymaking is not like evidence-based medicine, so how far should you go to bridge the divide between evidence and policy? Health Res Policy Syst. (2017) 15:2–11. 10.1186/s12961-017-0192-x 28446185 PMC5407004

[2] Oliver K Innvar S Lorenc T Woodman J. A systemic review of barriers to and facilitators of the use of evidence by policymakers. BMC Health Serv Res. (2014) 14:2. 10.1186/1472-6963-14-2 24383766 PMC3909454

[3] The Arc. Policy and Advocacy: Medicaid. (2021). Available online at: https://thearc.org/policy-advocacy/medicaid/#:~:text$=$For%20many%20people%20with%20intellectual,segregated%20nursing%20homes%20or%20institutions (accessed January 31, 2022).

[4] Larson SA Eschenbacher HJ Taylor B Pettingell S Sowers M Bourne ML. In-Home and Residential Long-Term Supports and Services For Persons With Intellectual or Developmental Disabilities: Status and Trends Through 2017. Minneapolis: University of Minnesota, Research and Training Center on Community Living, Institute on Community Integration (2020). Available online at: https://ici-s.umn.edu/files/aCHyYaFjMi/risp_2017.pdf (accessed February 1, 2022).

[5] Centers for Medicare and Medicaid Services. Instructions, Technical Guide and Review Criteria. (2019). Available online at: https://wms-mmdl.cms.gov/WMS/help/35/Instructions_TechnicalGuide_V3.6.pdf (accessed February 1, 2022).

[6] Lipson DJ. HCBS Quality Measures Issue Brief: Assessment and Care Planning Measures. Mathematica. (2019). Available online at: https://www.medicaid.gov/medicaid/quality-of-care/downloads/hcbs-quality- measures-brief-1-assessment-care-planning.pdf (accessed February 2, 2022).

[7] Academy Health. Meeting Highlights. Measuring the Quality of Home and Community-Based Services: A Conversation about Strategic Directions for Research and Policy. (2015). Available online at: https://www.thescanfoundation.org/sites/default/files/academyhealth_hcbs_quality_meeting_handout.pdf (accessed January 31, 2022).

[8] Harrington C Wiener JM Ross L Musumeci M. Key Issues In Long-Term Services and Supports Quality. Kaiser Family Foundation (2017). Available online at: https://www.kff.org/medicaid/issue-brief/key-issues-in-long-term-services-and-supports-quality/ (accessed January 30, 2022).

[9] Quinn K Weimar D Gray J Davies B. Thinking about clinical outcomes in medicaid. J Ambul Care Manage. (2016) 39:125. 10.1097/JAC.0000000000000130 26945295 PMC4851229

[10] National Quality Forum. Quality in Home and Community-Based Services to support community living: Addressing Gaps In Performance Measurement. (2016). Available online at: https://www .qualityforum.org/Publications/2016/09/Quality_in_Home_and_Community- Based_Services_to_Support_Community_Living__Addressing_Gaps_in_Per formance_Measurement.aspx (accessed January 30, 2022).

[11] Centers for Medicare and Medicaid Services. Request for Information: Recommended Measure Set for Medicaid-Funded Home and Community Based Services. (2020). Available online at: https://www.medic aid.gov/medicaid/quality-of-care/quality-improvement-initiatives/measuring- and-improving-quality-home-and-community-based-services-hcbs/index.html (accessed February 1, 2022).

[12] Bennett AD Curtis P Harrod CS. Bundling, Benchmarking, And Beyond: Paying for Value In Home-And Community-Based Services. (2018). Available online at: https://www.milbank.org/wp-content/uploads/2018/07/MMF-HCBS-Report-FINAL.pdf (accessed February 1, 2022).

[13] Centers for Medicare and Medicaid Services. Report to Congress: Identification of Quality Measurement Priorities –Strategic Plan, Initiatives, and Activities. (2019). Available online at: https://www.cms.gov/Medicare/Quality-Initiatives-Patient-Assessment-Instruments/QualityMeasures/Downloads/CMS-RTC-Quality-Measurement-March-1-2019_508 (accessed February 1, 2022).

[14] Schreiber M Duseja R Dahlerus C Suter L. The Future of Quality Measurement, 2020 and Beyond. Baltimore, MD: 2020 CMS Quality Conference (2018). Avaialble online at: https://www.cmsqualityconference.com/wp-content/uploads/2020/02/CMS-Quality-Conference-Agenda-2020_PRINT.pdf

[15] Potter DEB McKethan A Nguyen N. Long Term Care Quality Alliance Technical Appendices. (2008). Available online at: https://www.brookings.edu/wp-content/uploads/2012/04/Appendix.pdf (accessed February 1, 2022).

[16] Burnett J Seibert J. The Medicaid HCBS Experience Of Care Survey HCBS 2013. (accessed September 11, 2013).

[17] Centers for Medicare and Medicaid Services. Technical Assistance Guide For Administration Of The CAHPS® Home and Community-Based Services Survey. Available online at: https://www.medicaid.gov/medicaid/quality-of-care/downloads/hcbscahps-admin-ta-guide.pdf (accessed January 30, 2022).

[18] Friedman C. Building the Framework for IDD Quality Measures. Towson, Chicago, and Omaha: The Council on Quality and Leadership, the Institute for Public Policy for People with Disabilities, and Mosaic. (2018). Available online at: https://www.c-q-l.org/wp-content/uploads/2019/12/Building-The-Framework-For-IDD-Quality-Measures-2018.pdf (accessed January 30, 2022).

[19] Human Services Research Institute and National Association of State Directors of Developmental Disabilities Services. In Person Survey 2017-2018 Final Report. (2019). Available online at: https://www.nationalcoreindicators.org/upload/core-indicators/17-18_IPS_National_Report_PART_I_update_CA_entitlement.pdf (accessed January 30, 2022).

[20] National Association of State Directors of Developmental Disabilities Services and Human Services Research Institute. National Core Indicators Intellectual and Developmental Disabilities Measures Final Endorsement Announced by the National Quality Forum. (2022). Available online at: https://www.nasddds.org/wp-content/uploads/2022/01/Final-Press-release-NQF-_HSRI.pdf (accessed January 30, 2022).

[21] Council on Quality and Leadership (20). Personal Outcome Measures 2017. Measuring Outcomes Now and in the Future. Available online at: https://www.c-q-l.org/wp-content/uploads/2017/04/CQL-Personal-Outcome-Measures-Validation-Report-2017.pdf

[22] Thompson JR Bryant B Schalock RL Shogren KA Tassé MJ Wehmeyer ML. Supports Intensity Scale—Adult Version: User's Manual. Washington, DC: American Association on Intellectual and Developmental Disabilities (2015).

[23] Dinora P Bogenschutz M Broda M. Identifying predictors for enhanced outcomes for people with intellectual and developmental disabilities. Intellect Dev Disabil. (2020) 58:139–57. 10.1352/1934-9556-58.2.139 32240047

[24] Havercamp SM Krahn GL Larson SA Fujiura G Goode TD Kornblau BL. Identifying people with intellectual and developmental disabilities in national population surveys. Intellect Develop Disabil. (2019) 57:376–89. 10.1352/1934-9556-57.5.376 31568737

[25] Wagner JB Kim M Tassé MJ. Technology tools: Increasing our reach in national surveillance of intellectual and developmental disabilities. Intellect Dev Disabil. (2019) 57:463–75. 10.1352/1934-9556-57.5.463 31568740

[26] Bonardi A Krahn G Morris A, The National Workgroup on State and Local Health Data. Enriching Our Knowledge: State and Local Data to Inform Health Surveillance of the Population With Intellectual and Developmental Disabilities. (2019). Washington, DC: Administration on Intellectual and Developmental Disabilities. Available online at: https://doi-org.proxy.library.vcu.edu/10.1352/1934-9556-57.5.390

[27] Li C Tsoi EW Zhang AL Chen S Wang CJ. Psychometric properties of self-reported quality of life measures for people with intellectual disabilities: a systematic review. J Dev Phys Disabil. (2013) 25:253–70. 10.1007/s10882-012-9297-x

[28] Townsend-White C Pham ANT Vassos MV. A systematic review of quality of life measures for people with intellectual disabilities and challenging behaviours. J Intellect Disabil Res. (2012) 56:270–84. 10.1111/j.1365-2788.2011.01427.x 21679329

[29] Shogren KA. Considering context: an integrative concept for promoting outcomes in the intellectual disability field. Intellect Dev Disabil. (2013) 51:132–7. 10.1352/1934-9556-51.2.132 23537362

[30] Lakin KC Doljanac R Byun SY Stancliffe R Taub S Chiri G. Choice-making among Medicaid HCBS and ICF/MR recipients in six states. Am J Ment Retar. (2008) 113:325–42. 10.1352/2008.113.325-342 18702554

[31] Lu Z Cogan L McDermott S Lauer E Lindner S Tracy K. Disparities in diabetes management among Medicaid recipients with intellectual and developmental disabilities (IDD): Evidence from five US states. Disabil Health J. (2020) 13:100880. 10.1016/j.dhjo.2019.100880 31870791

[32] Houseworth J Stancliffe RJ Tichá R. Association of state-level and individual-level factors with choice making of individuals with intellectual and developmental disabilities. Res Dev Disabil. (2018) 83:77–90. 10.1016/j.ridd.2018.08.008 30144747

[33] Sannicandro T Parish SL Fournier S Mitra M Paiewonsky M. Employment, income, and SSI effects of postsecondary education for people with intellectual disability. Am J Intellect Dev Disabil. (2018) 123:412–25. 10.1352/1944-7558-123.5.412 30198768

[34] Mehling MH Tassé MJ. Impact of choice on social outcomes of adults with ASD. J Autism Dev Disorder. (2015) 5:1588–602. 10.1007/s10803-014-2312-6 25413143

[35] Neely-Barnes S Marcenko M Weber L. Does choice influence quality of life for people with mild intellectual disabilities?. Intellect Dev Disabil. (2008) 46:12–26. 10.1352/0047-6765(2008)46[12:DCIQOL]2.0.CO;2 18271608

[36] Bogenschutz M Broda M Lineberry S Prohn S. Testing a wellness indicators measure for people with intellectual and developmental disabilities. Dev Disabil Netw J. (2021) 2:85–10. 10.1352/2326-6988-10.1.19 35721389 PMC9201682

[37] Prohn S Dinora P Broda M Bogenschutz M Lineberry S. Measuring Four Personal Opportunities For Adults With Intellectual and Developmental Disabilities. Inclusion (2000). 10.1352/2326-6988-10.1.19 PMC9201575 35721258

[38] Lin E Balogh R Cobigo V Ouellette-Kuntz H Wilton A Lunsky Y. Using administrative health data to identify individuals with intellectual and developmental disabilities: a comparison of algorithms. J Intellect Disabil Res. (2013) 57:462–77. 10.1111/jir.12002 23116328

[39] Bertoncelli CM Altamura P Vieira ER Bertoncelli D Solla F. Using artificial intelligence to identify factors associated with autism spectrum disorder in adolescents with cerebral palsy. Neuropediatrics. (2019) 50:178–87. 10.1055/s-0039-1685525 31018221

[40] Maenner MJ Yeargin-Allsopp M Van Naarden Braun K Christensen DL Achieve LA. Development of a machine learning algorithm for the surveillance of autism spectrum disorder. PLoS ONE. (2016) 11:e0168224. 10.1371/journal.pone.0168224 28002438 PMC5176307

[41] Veale M Binns R. Fairer machine learning in the real world: mitigating discrimination without collecting sensitive data. Big Data Soc. (2017) 4:1–17. 10.1177/2053951717743530

[42] Broda M Bogenschutz M Dinora P Prohn S Lineberry S Ross E. Using machine learning to predict patterns of employment and day program participation. Am J Intellect Dev Disabil. (2021) 126:477–91. 10.1352/1944-7558-126.6.477 34700349

[43] Kaye HS Williamson J. Toward a model long-term services and supports system: state policy elements. Gerontologist. (2014) 54:754–61. 10.1093/geront/gnu013 24615231 PMC4163046

[44] Kaye HS Harrington C. Long-term services and supports in the community: toward a research agenda. Disabil Health J. (2015) 8:3–8. 10.1016/j.dhjo.2014.09.003 25445015

[45] Shogren KA Bradley VJ Gomez SC Yeager MH Schalock RL Borthwick-Duffy S. Public policy and the enhancement of desired outcomes for persons with intellectual disability. Intellect Dev Disabil. (2009) 47:307–19. 10.1352/1934-9556-47.4.307 19650684

[46] Gilson CB Bethune LK Carter EW McMillan ED. Informing and equipping parents of people with intellectual and developmental disabilities. Intellect Dev Disabil. (2017) 55:347–60. 10.1352/1934-9556-55.5.347 28972871

[47] Lavis JN Robertson D Woodside JM McLeod CB Abelson J. How can research organizations more effectively transfer research knowledge to decision makers? Milbank Q. (2003) 81:221–48. 10.1111/1468-0009.t01-1-00052 12841049 PMC2690219

[48] Terras MM Jarrett D McGregor SA. The importance of accessible information in promoting the inclusion of people with an intellectual disability. Disabilities. (2021) 1:132–50. 10.3390/disabilities1030011

[49] Waight M Oldreive W. Investigating accessible information formats with people who have learning disabilities. Learn Disabil Pract. (2021) 24:23–30. 10.7748/ldp.2020.e2031

[50] Van Eperen L Marincola FM. How scientists use social media to communicate their research. J Transl Med. (2011) 9:1–3. 10.1186/1479-5876-9-199 22085450 PMC3231985

[51] Pew Research Center. Social Media Use in 2021. (2021). Available online at: https://www.pewresearch.org/internet/2021/04/07/social-media-use-in-2021/

[52] Ramsten C Martin L Dag M Hammar LM. Information and communication technology use in daily life among young adults with mild-to-moderate intellectual disability. J Intellect Disabil. (2020) 24:289–308. 10.1177/1744629518784351 30010467

[53] Shpigelman CN Gill CJ. How do adults with intellectual disabilities use Facebook? Disabil Soc. (2014) 29:1601–16. 10.1080/09687599.2014.966186

[54] Caton S Chapman M. The use of social media and people with intellectual disability: a systematic review and thematic analysis. J Intellect Dev Disabil. (2016) 41:125–39. 10.3109/13668250.2016.1153052

[55] Arnautu D Dagenais C. Use and effectiveness of policy briefs as a knowledge transfer tool: a scoping review. Human Soc Sci Commun.(2021) 8:1–14. 10.1057/s41599-021-00885-9

[56] Arcury TA Wiggins MF Brooke C Jensen A Summers P Mora DC. Using “policy briefs” to present scientific results of CBPR: farmworkers in North Carolina. Prog Commun Health Partner. (2017) 11:137–47. 10.1353/cpr.2017.0018 28736406 PMC6262882

[57] Chinn D Homeyard C. Easy read and accessible information for people with intellectual disabilities: is it worth it? a meta-narrative literature review. Health Expect. (2017) 20:1189–200. 10.1111/hex.12520 27862757 PMC5689240

[58] Mmatli TO. Translating disability-related research into evidence-based advocacy: the role of people with disabilities. Disabil Rehabil. (2009) 31:14-22. 10.1080/09638280802280387 18946807

[59] Coons KD Watson SL. Conducting research with individuals who have intellectual disabilities: Ethical and practical implications for qualitative research. J Dev Disabil. (2013) 19:14.

[60] Feldman MA Bosett J Collet C Burnham-Riosa P. Where are persons with intellectual disabilities in medical research? a survey of published clinical trials. J Intellect Disabil Res. (2014) 58:800–9. 10.1111/jir.12091 24001184

[61] Rios D Magasi S Novak C Harniss M. Conducting accessible research: including people with disabilities in public health, epidemiological, and outcomes studies. Am J Public Health. (2016) 106:2137–44. 10.2105/AJPH.2016.303448 27736212 PMC5104996

[62] O'Brien P McConkey R García-Iriarte E. Co-researching with people who have intellectual disabilities: Insights from a national survey. J Appl Res Intellect Disabil. (2014) 27:65–75. 10.1111/jar.12074 24376031

[63] Williamson HJ van Heumen L Schwartz AE. Photovoice with individuals with intellectual and/or developmental disabilities: lessons learned from inclusive research efforts. J Commun Based Res Pract. (2020) 3:8. 10.33596/coll.45

[64] Bigby C Frawley P. Reflections on doing inclusive research in the “Making Life Good in the Community” study. J Intellect Dev Disab. (2010) 35:53–61. 10.3109/13668251003716425 20560691

[65] Bigby C Frawley P Ramcharan P. Conceptualizing inclusive research with people with intellectual disability. J Appl Res Intellect Disabil. (2014) 27:3–12. 10.1111/jar.12083 24390972

[66] Conder J Milner P Mirfin-Veitch B. Reflections on a participatory project: the rewards and challenges for the lead researchers. J Intellect Dev Disabil. (2011) 36:39–48. 10.3109/13668250.2010.548753 21309660

[67] Fadlallah R El-Jardali F Monier M Hemadi N Arif K Langlois E. Using narrative to impact health policy-making: a systematic review. Health Res Policy Syst. (2019) 17:26. 10.1186/s12961-019-0423-4 30836972 PMC6402129

